# Repression of Mitochondrial Translation, Respiration and a Metabolic Cycle-Regulated Gene, *SLF1*, by the Yeast Pumilio-Family Protein Puf3p

**DOI:** 10.1371/journal.pone.0020441

**Published:** 2011-05-31

**Authors:** Marc Chatenay-Lapointe, Gerald S. Shadel

**Affiliations:** 1 Department of Pathology, Yale University School of Medicine, Yale University, New Haven, Connecticut, United States of America; 2 Department of Genetics, Yale University School of Medicine, Yale University, New Haven, Connecticut, United States of America; University of Medicine and Dentistry of New Jersey, United States of America

## Abstract

Synthesis and assembly of the mitochondrial oxidative phosphorylation (OXPHOS) system requires genes located both in the nuclear and mitochondrial genomes, but how gene expression is coordinated between these two compartments is not fully understood. One level of control is through regulated expression mitochondrial ribosomal proteins and other factors required for mitochondrial translation and OXPHOS assembly, which are all products of nuclear genes that are subsequently imported into mitochondria. Interestingly, this cadre of genes in budding yeast has in common a 3′-UTR element that is bound by the Pumilio family protein, Puf3p, and is coordinately regulated under many conditions, including during the yeast metabolic cycle. Multiple functions have been assigned to Puf3p, including promoting mRNA degradation, localizing nucleus-encoded mitochondrial transcripts to the outer mitochondrial membrane, and facilitating mitochondria-cytoskeletal interactions and motility. Here we show that Puf3p has a general repressive effect on mitochondrial OXPHOS abundance, translation, and respiration that does not involve changes in overall mitochondrial biogenesis and largely independent of TORC1-mitochondrial signaling. We also identified the cytoplasmic translation factor Slf1p as yeast metabolic cycle-regulated gene that is repressed by Puf3p at the post-transcriptional level and promotes respiration and extension of yeast chronological life span when over-expressed. Altogether, these results should facilitate future studies on which of the many functions of Puf3p is most relevant for regulating mitochondrial gene expression and the role of nuclear-mitochondrial communication in aging and longevity.

## Introduction

The assembly of the mitochondrial oxidative phosphorylation (OXPHOS) system is a remarkably complex process, in part, because expression from two genomes, mtDNA and nuclear DNA, must be coordinated [1]. Furthermore, this process is more intricate given the fact that all of proteins required for replication of mtDNA, as well as transcription and translation of it mRNA products, are encoded by nuclear DNA and imported into the organelle [2], [3]. The budding yeast, *Saccharomyces cerevisiae*, has been used extensively as a model system to identify and understand signaling pathways that mediate this nuclear-mitochondrial crosstalk [1], [4], [5], [6], [7]. However, even in yeast, these are far from understood and the role of post-transcriptional events as a means of regulating nuclear-encoded mitochondrial gene expression has not been studied to the same extent as transcriptional regulatory circuits.

Budding yeast can respond quickly to changes in nutrient levels by modulating protein expression in both space and time. For example, glucose-repressed genes are regulated by both transcriptional and post-transcriptional mechanisms at the level of mRNA [4], [5]. The nuclear genes that encode mitochondrial ribosome proteins (MRPs) are co-regulated as determined in numerous microarray studies [8]. However, there is lack of lack of obvious transcriptional regulatory elements in the promoters of the genes in this module [9] and it has been postulated that these have been lost during the evolution of glucose-repressed yeasts [10]. Alternatively, 73 of the 74 nucleus-encoded MRP genes possess a UGUANAUA motif in their 3′-UTR that likely directs their co-regulation including during the yeast metabolic cycle [11]. In fact, almost all transcripts encoding for factors involved with mitochondrial protein synthesis or assembly of the mitochondrial OXPHOS system have this Puf3 Element (P3E) [12], [13], which is bound by Puf3p [14] to stimulate the decapping and deadenylation of P3E-containing transcripts [15]. The repression by the PUF family of proteins is conserved and involves the binding of Pop2p, which in turn recruits CCR4-NOT deadenylase machinery as well as decapping factors, Dcp1p and Dhh1p [16].

Early work by Butow and colleagues showed that cytoplasmic ribosomes are attached to the mitochondrial outer membrane [17], strongly suggesting co-translational import of polypeptides into mitochondria [18]. In yeast, many nucleus-derived transcripts encoding mitochondrial proteins are localized to mitochondrial-bound ribosomes via information residing in their 3′-UTR [19]. The mRNA localization phenomenon is apparently conserved, as human MrpS12 mRNA associates with mitochondria in a 3′-UTR dependent fashion [20]. In addition to its role in promoting mRNA decay, Puf3p also is important for mitochondrial transcript localization. That is, mitochondrial association of the majority of P3E transcripts is reduced or abrogated in the absence of Puf3p [18]. The mitochondrial localization of transcripts by Puf3p is likely linked to import of the polypeptide it encodes, as genetic interactions exist between *PUF3* and *TOM20* [21], a component of the TOM complex responsible for translocation of polypeptides across the outer membrane. Thus, Puf3p appears to be involved in controlling the logistics of OXPHOS assembly and may do so spatially and in response to the yeast metabolic cycle [22]. There is also some indication that the activity of Puf3p in mRNA decay are regulated by the nutrient-sensing TORC1 signaling pathway [12]. Finally, Puf3p physically links the mitochore to the actin cytoskeleton, allowing for bud-directed organellar movement during cell division [23] and is associated with P bodies [24]. With so many assigned functions and locations, how Puf3p operates to control various cellular processes remains unclear, although models have begun to formulate [13], [25]. The goal of this study was to gain additional insight into how Puf3p controls mitochondrial gene expression and respiration and its relationship to the TORC1 pathway, which we have shown represses mitochondrial translation and curtails yeast chronological life span [26], [27].

## Results

### Puf3p represses mitochondrial translation, OXPHOS subunit accumulation and respiration without affecting overall mitochondrial biogenesis

**Figure 1:**
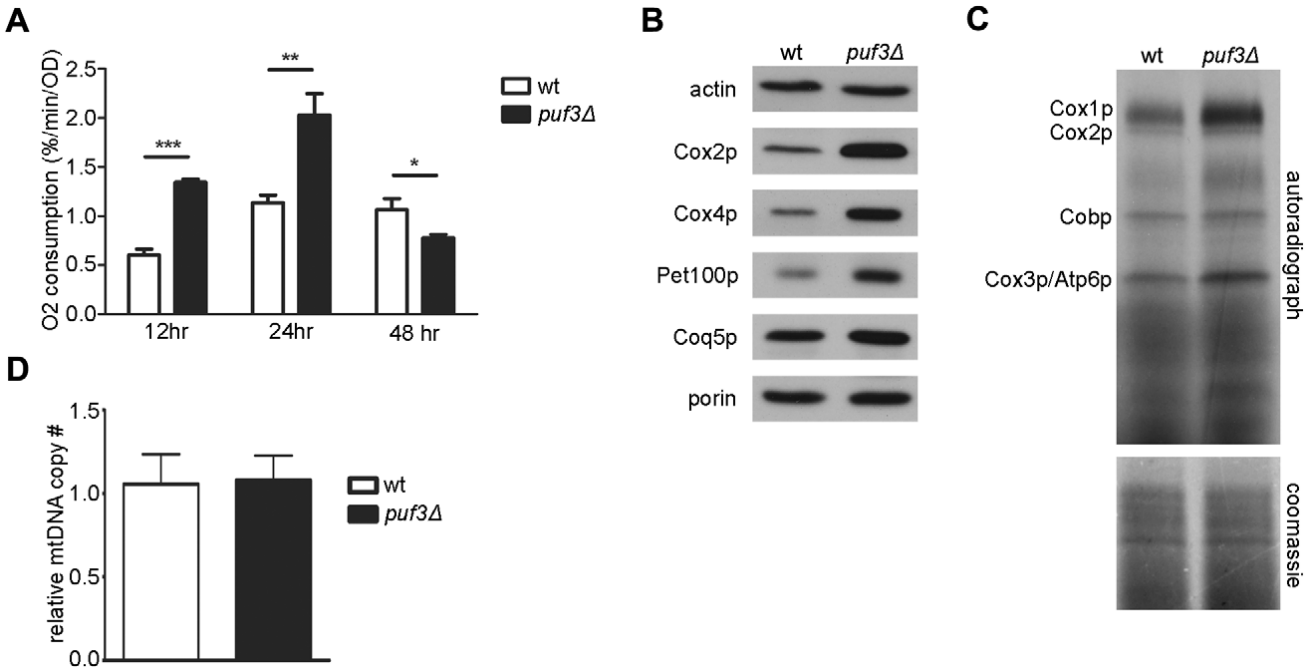
Analysis of *puf3Δ* Yeast Strains. Analysis of wild-type (wt) and an isogenic *puf3Δ* strains are shown. (A) Mitochondrial oxygen consumption assayed at 12 hours (log phase), 24 hours (early stationary phase), and 48 hours post-inoculation (1 day into stationary phase). The bars represent the mean ± SD with p-values as indicated (*<0.05, **<0.01, ***<0.001). Statistical analysis was performed with Prism 5 software, using a Student's t-test (unpaired, two-tailed). (B) Western blot of indicated proteins in log-phase growth (representative of three biological replicates). (C) Autoradiogram of separated mtDNA-encoded proteins labeled with ^35^S-methionine and ^35^S -cysteine at logarithmic growth. A section of the same gel stained with coomassie blue to demonstrate loading is shown underneath. (D) mtDNA copy number.

We analyzed multiple mitochondrial parameters in wild-type (DBY2006) and isogenic *puf3Δ* yeast strains grown in synthetic dextrose medium. Lack of Puf3p increased mitochondrial oxygen consumption during logarithmic growth and at early stationary phase time points, however, this was not sustained (and even decreased slightly) later in stationary phase (Figure 1A). The increase in mitochondrial respiration observed in log-phase growth was accompanied by a corresponding robust increases in the steady-state levels of multiple mitochondrial proteins, including Cox2p and Cox4p, mtDNA-encoded and nucleus-encoded OXPHOS subunits, respectively (Figure 1B) and Pet100p, a nucleus-encoded protein with a Puf3 element in its 3′ UTR. We observed no significant increases in “mitochondrial housekeeping” markers Coq5p and porin compared to the actin loading control, suggesting that overall mitochondrial biogenesis was not increased in *puf3Δ* cells (Figure 1B). There was also no increase in mtDNA per cell in *puf3Δ* strains, which is consistent with mitochondrial biogenesis not being altered (Figure 1D). Finally, there was an apparently global up-regulation of mitochondrial translation in *puf3Δ* strains compared to the wild-type control during log-phase growth (Figure 1C). The up-regulation of mitochondrial translation and OXPHOS subunits per se (i.e. without a corresponding increase in overall mitochondrial biogenesis) is consistent with Puf3p being a negative regulator of the subset of nucleus-encoded genes involved in mitochondrial translation and OXPHOS assembly [23], but not overall organelle biogenesis.

**Figure 2:**
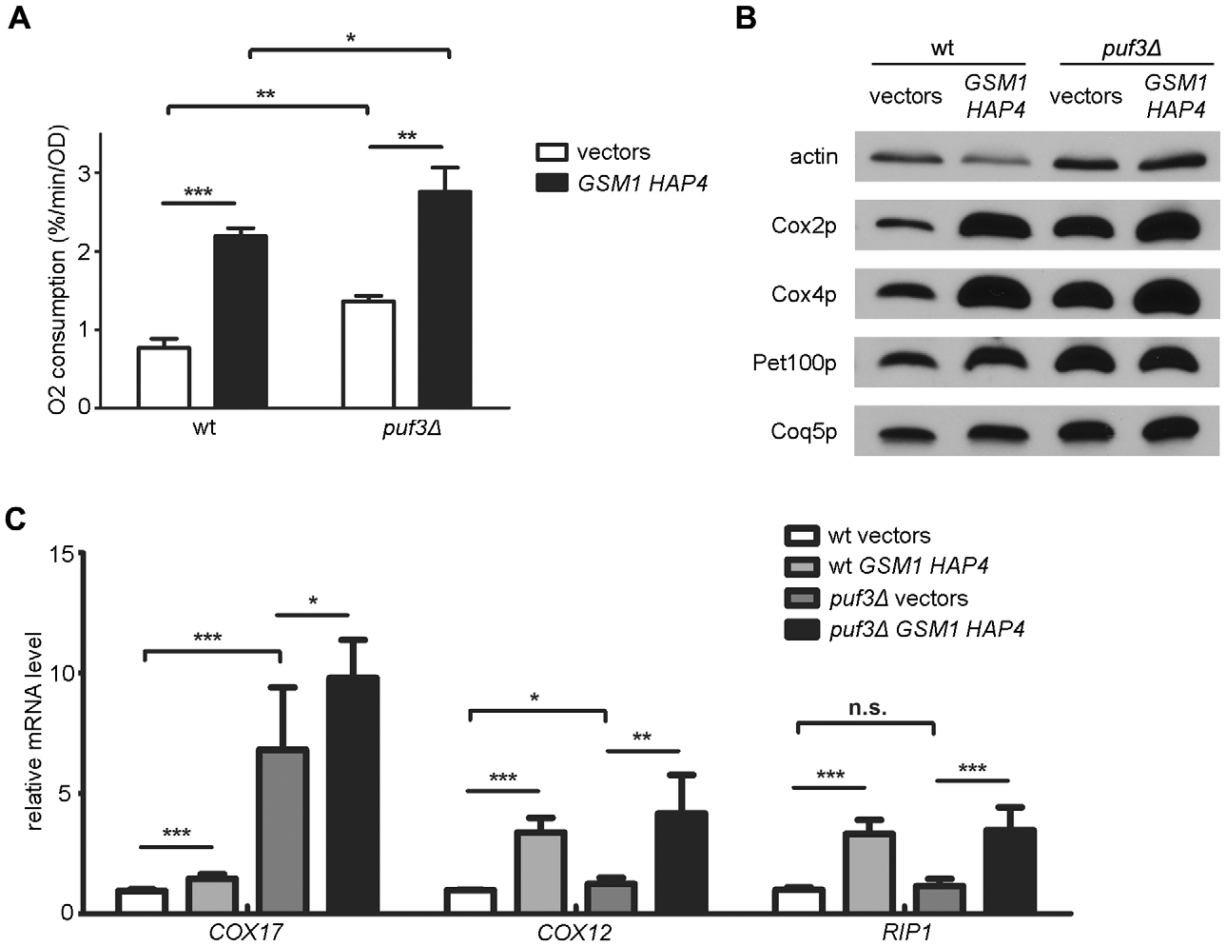
The Regulation of Respiration by Puf3p is Independent of Mitochondrial Biogenesis Pathways. Shown is the analysis of wild-type (wt) and *puf3Δ* strains with plasmids over-expressing *HAP4* and *GSM1* (*HAP4 GSM1*) and corresponding empty vectors (vectors) (A) Mitochondrial oxygen consumption during late-log phase of growth. (B) Western blot analysis (representative of three biological replicates). (C) Steady-state levels of *COX17*, *COX12*, and *RIP1* transcripts in late log phase of growth. The values indicate the mean +/− SD with p-values indicated as described in the legend of Figure 1.

To probe further whether up-regulation of mitochondrial respiration in *puf3Δ* strains was independent of mitochondrial biogenesis pathways, we over-expressed Hap4p, a known transcriptional regulator of mitochondrial biogenesis, and Gsm1p, another transcription factor implicated in regulating the expression of OXPHOS proteins [28], [29], in wild-type and *puf3Δ* strains. Simultaneous over-expression of these two transcription factors caused similar increases in respiration in both wild-type and *puf3Δ* strains (Figure 2A) and, further increases in the already heightened steady-state levels of Cox2p and Cox4p in the *puf3Δ* strain (Figure 2B). Finally, we assayed the steady-state levels of *COX17* transcripts, a canonical Puf3 target [15], and two non-P3E-target transcripts, *RIP1* and *COX12*, by quantitative real-time RT-PCR. As expected, deletion of *PUF3* leads to a dramatic increase in *COX17* transcripts, but had little or no effect on the non-P3E targets, while over-expression of *HAP4* and *GSM1* in a wild-type strain led to an increase in *COX12* and *RIP1* mRNA (Figure 2C). Importantly, Hap4p/Gsm1p over-expression increased all of these transcripts to a similar degree in the *puf3Δ* strain (Figure 2C), bolstering our conclusion that up-regulation of respiration in the absence of Puf3p is largely independent of pathways that increase mitochondrial biogenesis.

### Over-expression of Puf3p Reduces Mitochondrial Respiration and Steady-state levels of OXPHOS-related Proteins in Late Log-phase Cultures

**Figure 3:**
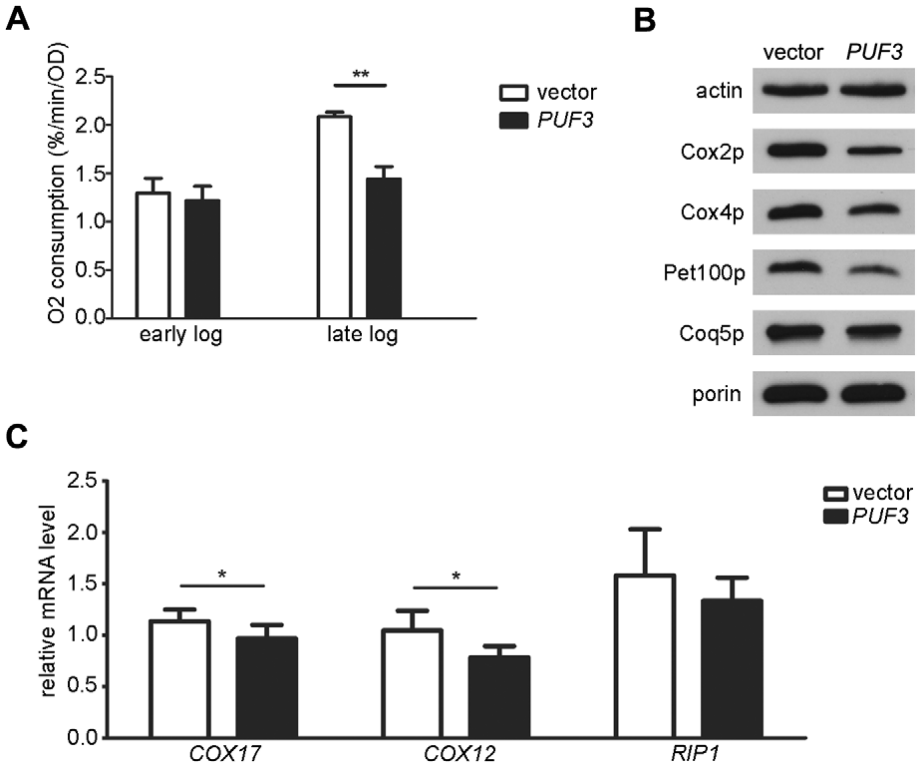
Analysis in strains that over-express Puf3p. Shown is a wild-type strain with a plasmid that over-expresses Puf3p (*PUF3*) or the corresponding empty vector (vector). (A) Mitochondrial oxygen consumption during early and late log phase. (B) Western blot analysis of protein extracts derived from late log cultures (representative of three biological replicates). (C) Steady-state levels of *COX17*, *COX12*, and *RIP1* transcripts in late log phase. The values indicate the mean +/− SD with p-values indicated as described in the legend of Figure 1.

Unlike deletion of *PUF3*, which increased mitochondrial oxygen consumption and OXPHOS protein accumulation during log-phase growth, over-expression of *PUF3* did not significantly repress these parameters until later in log phase (Figure 3A and 3B) when cells have already undergone the diauxic shift and significantly increased mitochondrial biogenesis and respiration. Only a modest decline in the Puf3p-target transcript *COX17* was observed under these conditions and the two other mitochondrial transcripts analyzed were either minimally effected or not at all (Figure 3C). These data suggest that the repression of respiration by Puf3p over-expression may involve functions other than mRNA transcript decay.

### Over-expression of *PUF3* Curtails Life Span Extension in a *tor1Δ* strain, but is Not a Major Mediator of the Increased Respiration Due to Reduced mTORC1 signaling

**Figure 4:**
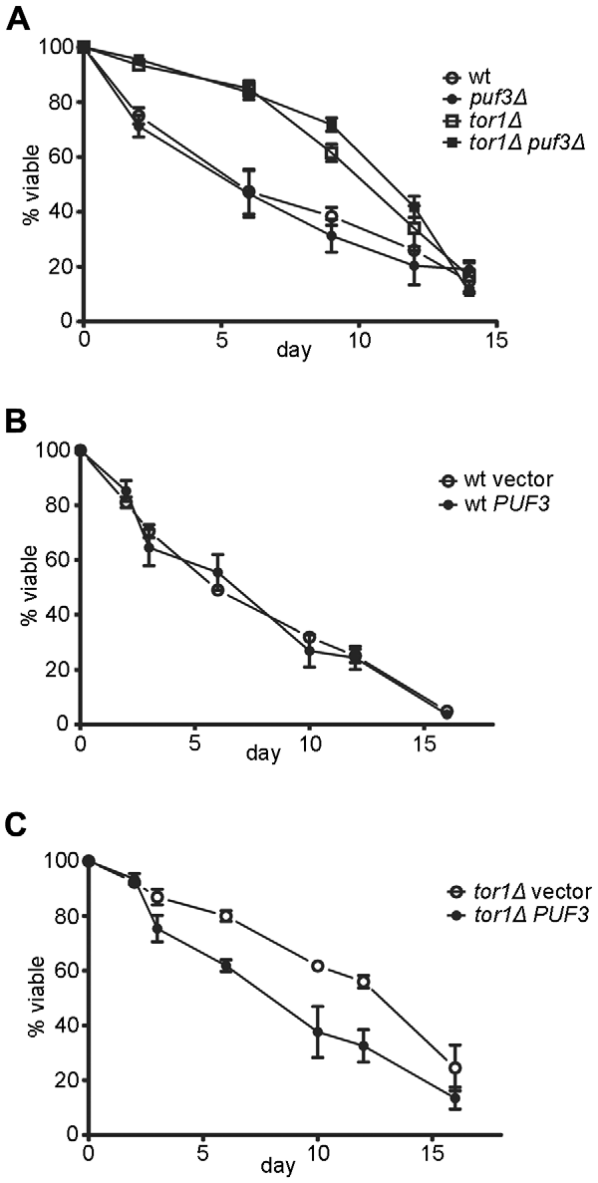
Chronological Aging in strains that lack or over-express Puf3p. Shown are chronological life span curves (viability as a function of time in days of stationary phase) of wild-type strain compared to isogenic *puf3Δ*, *tor1Δ,* and *puf3Δ tor1Δ* strains (A), a wild-type strain with an empty vector or a plasmid that over-expresses *PUF3* (B), or a *tor1Δ* strain with an empty vector or a plasmid that over-expresses *PUF3* (C). Each point is the mean +/− SD of three biological replicates.

Reactive oxygen species (ROS) are produced during metabolism and are thought to contribute to aging. For example, they are generated by the mitochondrial electron transport chain, placing these organelles at the nexus of oxidative stress and aging according to the mitochondrial theory of aging. We found previously [26], [27] that reduced TORC1 signaling increases the density of OXPHOS complexes in a manner that reduces ROS in stationary phase and extends yeast chronological life span. Given that similar changes in OXPHOS abundance and respiration are observed in a *puf3Δ* strain (Figure 1), we ascertained whether Puf3p is involved in regulating CLS and/or is a downstream effector of TORC1 effects on mitochondrial function. However, deletion of *PUF3* in wild-type or *tor1Δ* strains did not affect CLS (Figure 4A). Furthermore, no difference in CLS were observed in a wild-type strain that over-expresses Puf3p (Figure 4B). However, over-expression of Puf3p did partially reduce the extended CLS of a *tor1Δ* strain (Figure 4C).

**Figure 5:**
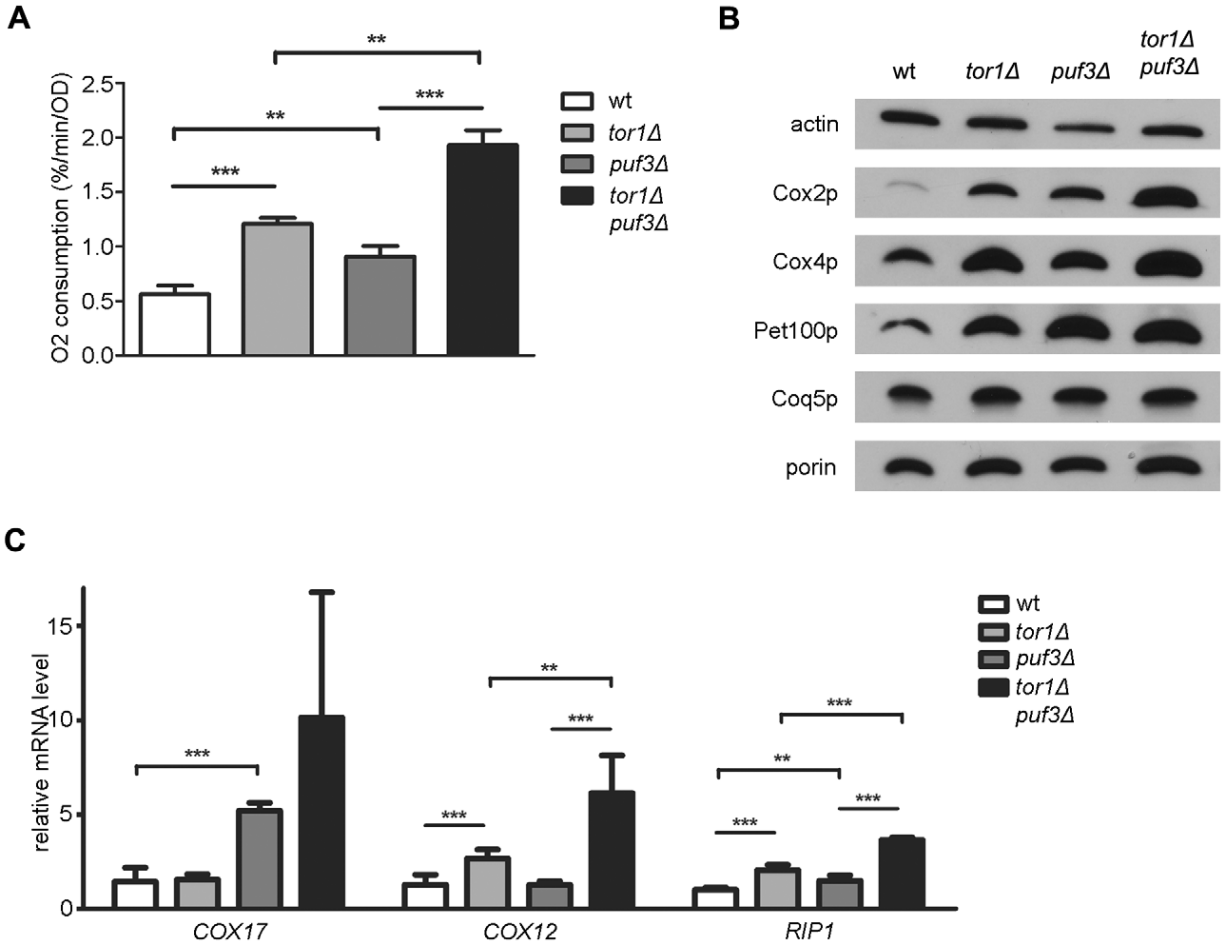
Comparison of mitochondrial parameters in *puf3Δ*, *tor1Δ,* and *puf3Δ tor1Δ* mutant strains. (A) Mitochondrial oxygen consumption during early log phase. (B) Western blot analysis of protein extracts derived from log phase cultures (representative of three biological replicates). (C) Steady-state levels of *COX17*, *COX12*, and *RIP1* transcripts in log phase. The values indicate the mean +/− SD with p-values indicated as described in the legend of Figure 1.

**Figure 6:**
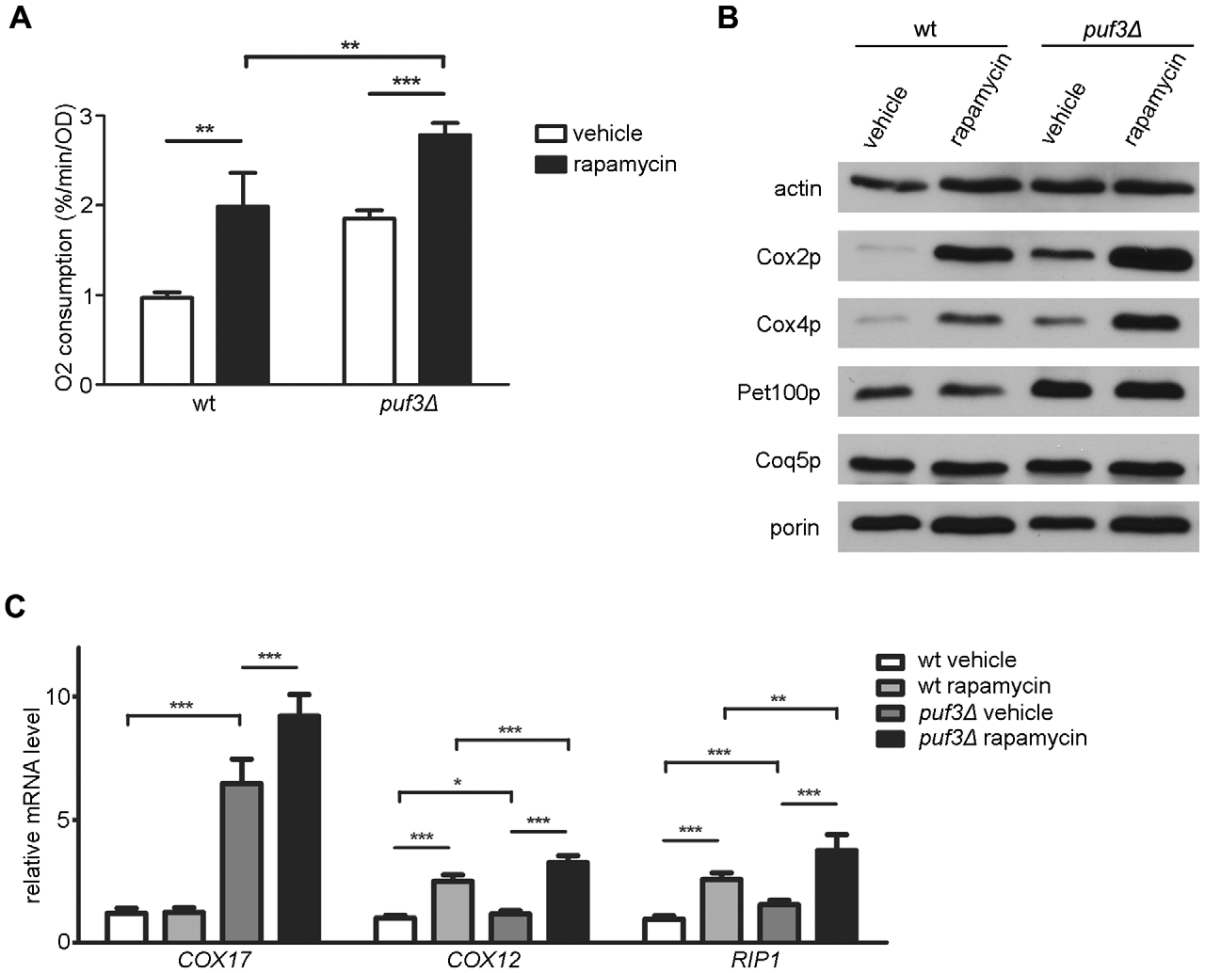
Comparison of mitochondrial parameters in wild-type and *puf3Δ* mutant strains treated with the TORC1 inhibitor rapamycin. (A) Mitochondrial oxygen consumption of cells treated with rapamycin during early log phase and analyzed during late log phase. (B) Western blot analysis of protein extracts derived from late log phase cultures (representative of three biological replicates). (C) Steady-state levels of *COX17*, *COX12*, and *RIP1* transcripts in late log phase. The values indicate the mean +/− SD with p-values indicated as described in the legend of Figure 1.

To probe more directly the potential involvement of Puf3p in the regulation of mitochondrial function by TORC1, we compared mitochondrial parameters in *tor1Δ* and *puf3Δ* single-mutant strains to those in a *tor1Δ puf3Δ* double-mutant strain. While both single-mutant strains had increased mitochondrial oxygen consumption and Cox2p and Cox4p levels compared to the isogenic wild-type strains, the double-mutant strains displayed an additive increase in these parameters (Figure 5A and 5B). In contrast to Cox2p and Cox4p, Pet100p (a Puf3p target) did not display an additive increase in the double-mutant strain (Figure 5B). Comparison of the “mitochondrial housekeeping” proteins Coq5p and porin to the loading control actin revealed only modest changes in overall mitochondrial abundance in any of the mutant strains compared to the wild-type control (Figure 5B). Finally, analysis of mitochondrial transcripts in this cadre of strains revealed complex regulation. While loss of Puf3p led to increased levels of the Puf3-target *COX17* transcript, this was enhanced further by loss of Tor1p (Figure 5C, left). Similarly, loss of Tor1p resulted in increased amounts of *COX12* and *RIP1* transcripts, but this too was enhanced further (in an apparently additive manner) by lack of Puf3p (Figure 5C, center and right). Similar results with regard to respiration, steady-state mitochondrial marker protein levels, and target transcripts were observed in wild-type and *puf3Δ* strains treated with rapamycin (Figure 6). These data indicate that, while there might be some degree of crosstalk/synergism between the Puf3p and TORC1 pathways with regard to specific mitochondrial targets and effects, these pathways operate largely independently with regard to respiration and CLS.

### Over-expression of Slf1p Increases Mitochondrial Respiration and Chronological Life Span

**Figure 7:**
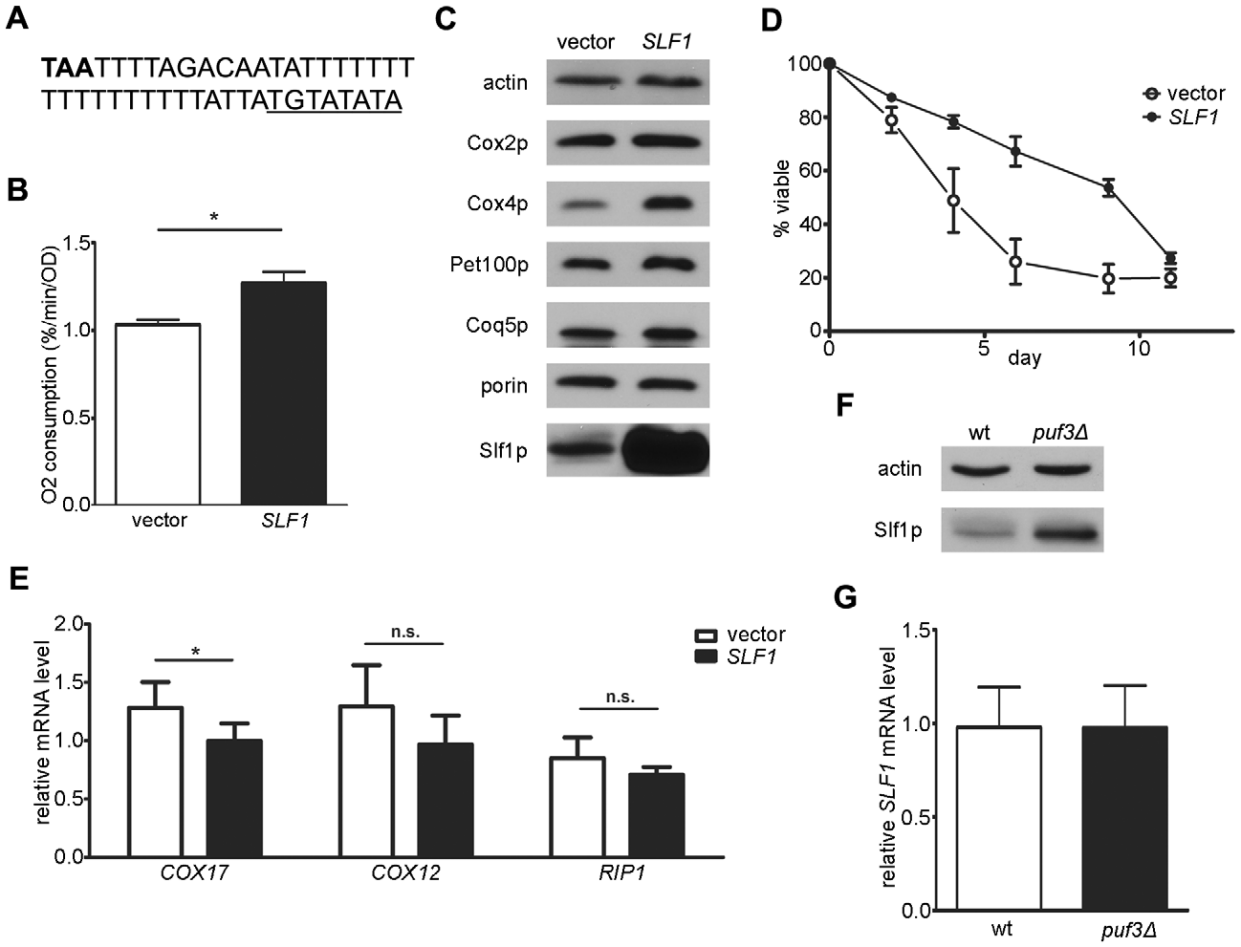
Over-expression of Slf1p increases respiration and CLS, and expression of Sls1p is repressed by Puf3p. (A) *SLF1* DNA sequence depicting the stop codon (bold) and Puf3 element (underlined). (B) Mitochondrial oxygen consumption of wild-type cells carrying an empty vector (YEp351) or YEp351 with *SLF1* during early stationary phase. (C) Western blot analysis of protein extracts derived from the same strains in (B) (representative of three biological replicates). (D) CLS analysis of the same strains in (B). (E) Q-RT-PCR analysis *COX17*, *COX12*, and *RIP1* transcript levels at early stationary phase from the same strains in (B). Analysis of Slf1p levels by western blot (F) and *SLF1* mRNA levels by RT-PCR (G) in wild-type and *puf3Δ* strains in log-phase growth (representative of three biological replicates).

The vast majority of genes in the Puf3 regulon are regulators of mitochondrial translation (e.g. mitochondrial ribosomal proteins and translation factors) or processes immediately downstream (e.g. OXPHOS assembly factors) that control respiration. To find additional genes potentially involved in mitochondrial function, others identified transcripts that were both localized to the mitochondrial periphery and clustered with P3E mRNAs as a function of the yeast metabolic cycle (YMC) [22]. We hypothesized that a subset of the Puf3 regulon is not involved with mitochondrial translation directly, but may nonetheless be important for mitochondrial respiration. To test this hypothesis, we queried the YMC microarray database SCEPTRANS [30] for mRNAs that displayed a YMC pattern of expression, but are not predicted to localize to mitochondria. Of the top 50 transcripts coregulated with *COX17* in the YMC, three met this criterion: *YOR248W*, a dubious ORF; *SRL1*, a mannoprotein associated with the cell wall [31]; and *SLF1*, and RNA-binding protein associated with cytoplasmic polysomes [32]. A consensus P3E exists shortly after the stop codon in the *SLF1* mRNA (Figure 7A), consistent with it being a target of Puf3p. Thus Slf1p seemed like a likely candidate factor that could potentially regulate respiration and was studied further. While *slf1Δ* strains did not reveal any obvious growth or respiration phenotype in fermentable or non-fermentable media, over-expression of Slf1p resulted in a modest, but reproducible increase in mitochondrial oxygen consumption (Figure 7B), a pronounced increase in Cox4p, and modest increases in Cox2p and Pet100p (Figure 7C). Over-expression of Slf1p also significantly increased median CLS (Figure 7D) and significantly reduced the amount of the Puf3-target transcript, *COX17*, while having minimal or no effect on non-puf3-target transcripts (Figure 7E). Finally, Slf1p protein (Figure 7F), but not its transcript (Figure 7G), was up-regulated in a *puf3Δ* strain. Altogether, these data indicate that Slf1p a P3E-containing gene that encodes a putative regulator of mitochondrial function and CLS and is repressed by Puf3p at the post-transcriptional level.

## Discussion

The pumilio family (PUF) of proteins in yeast binds distinct sets transcripts and regulates their stability, translation and subcellular localization [25]. In this study, we have examined Puf3p, the member of this family that preferentially binds to nuclear mRNAs that encode proteins involved in mitochondrial translation and OXPHOS assembly [22]. Based on our results, we conclude that 1) Puf3p has a general repressive effect on mitochondrial translation, OXPHOS protein accumulation, and respiration, 2) the Puf3p repressive effect on respiration is mediated by changes in mitochondrial OXPHOS complex density, as opposed to modulating overall mitochondrial biogenesis, 3) that effects of Puf3p on respiration are largely independent of the TORC1 signaling pathway, and 4) that Slf1p is member of the Puf3 regulon that is repressed by Puf3p at the post-transcriptional level and putatively regulates mitochondrial respiration, possibly by influencing cytoplasmic translation of nuclear transcripts that encode mitochondrial proteins. We will also discuss the relevance of effects we observed of over-expression of Puf3p or Slf1p on chronological aging. Altogether, these results should facilitate future studies on which of the many functions of Puf3p is most relevant for regulating mitochondrial gene expression and function.

Our results indicate that, despite its postulated pro-mitochondrial functions of localizing transcripts to the mitochondrial periphery [18] and promoting mitochondrial movement [23], Puf3p is an overall negative regulator of mitochondrial translation and respiration (Figures 1 and 3). This repressive effect on respiration appears to be primarily manifest at the level OXPHOS system abundance as opposed to changes in overall mitochondrial biogenesis as lack of Puf3p increases translation of mtDNA-encoded OXPHOS subunits and steady-state levels of mtDNA-encoded and nuclear-DNA encoded OXPHOS proteins without increasing mitochondrial housekeeping markers or mtDNA (Figure 1). This conclusion is bolstered by similar, but opposite effects observed on respiration and OXPHOS density upon over-expression of Puf3p (Figure 3) and additive effects of over-expression of Hap4p and Gsm1p on these parameters in a *puf3Δ* strain (Figure 2). These results fully corroborate that the main cadre of transcripts regulated by Puf3p encodes mitochondrial ribosomal proteins and other factors involved in mitochondrial translation and OXPHOS assembly, which does not include genes needed for wholesale organelle biogenesis. At this point we can only speculate on which function of Puf3p is dominating the repressive effects on respiration. At first glance, this may seem contrary to the idea that Puf3p is needed to localize nuclear transcripts to mitochondrial outer membrane for co-translational import of the mitochondrial proteins they encode. However, loss of this function in *puf3Δ* strains may be compensated for by the overall increase in P3E transcript abundance and/or their release from a translationally repressive condition such as localization to P bodies [24]. That over-expression of Puf3p had little impact on *COX17* transcript abundance (Figure 3C) also suggests that functions of Puf3p other than degradation of P3E-target transcripts per se are mediating the repressive effect on respiration under this circumstance.

The TORC1 inhibitor rapamycin causes the collective up-regulation of P3E transcripts in yeast and slows the decay of a reporter transcript bearing a 3′-UTR P3E [12]. This, coupled with the involvement of TORC1 pathway in regulating the deadenylation and decapping of transcripts during the diauxic shift [33], when mitochondrial respiration is up-regulated, suggested that Puf3p may regulated by TORC1 signaling. This hypothesis appeared even more likely when we observed that deletion of *PUF3* resulted in up-regulation of OXPHOS density without an increase in overall mitochondrial biogenesis, which is a phenotype we had documented previously in *tor1Δ* and *sch9Δ* strains that have reduced TORC1 signaling [27]. Despite these intriguing connections, the simplest interpretation of our results comparing mitochondrial parameters in a *tor1Δ puf3Δ* double-mutant strain to the respective single-mutant strains (Figure 5) is that Puf3p is not a major downstream effector of TORC1 with regard to repressing mitochondrial respiration. However, our results do indicate some degree of interplay between TORC1 and Puf3p regulation. For example, an epistatic relationship was apparent with regard to the steady-state levels of Pet100p (Figure 5B), whose mRNA has a P3E element, which might indicate that TORC1 and Puf3p regulation may indeed converge on certain P3E-containing transcripts. Furthermore, there were synergistic (more than additive) increases in *COX17* and *COX12* transcripts in the *tor1Δ puf3Δ* double-mutant strain (Figure 5C), consistent with crosstalk between the Puf3p and TORC1 pathways. Recently, the mitochondrial protein Fmp48p was found in a global screen to interact with both Tor1p and Puf3p [34], which is intriguing with regard to potential crosstalk between these pathways.

We identified Slf1p as a new factor involved, directly or indirectly, in mitochondrial regulation based on its co-regulation in the yeast metabolic cycle with other Puf3 targets (Figure 7). Since this protein is most likely involved in cytoplasmic translation [32], it is one of the minority of Puf3 targets that apparently does not affect mitochondrial translation or OXPHOS assembly in the organelle directly. Over-expression of Slf1p modestly increases respiration (Figure 7B), significantly increases Cox4p levels, and modestly increases Cox2p and Pet100p (Figure 7C). It also decreases *COX17* transcripts, but not two other non-P3E targets (Figure 7E). These results indicate that this protein is likely involved in regulating Puf3-target transcripts, but may be somewhat selective. Finally, even though the *SLF1* transcript contains a predicted P3E element (Figure 7A), its abundance is not affected by deletion of *PUF3* (Figure 7G). However, Slf1p is up-regulated in the *puf3Δ* strain (Figure 7F). Therefore, Slf1p is a unique member of the Puf3p mitochondrial regulon that is itself regulated by Puf3p at the post-transcriptional level. Slf1p binds RNA, localizes to cytoplasmic polysomes, and influences sensitivity to translational inhibitors [32]. In addition, four out of seven high-throughput physical associations of Slf1 are with factors regulating mRNAs and translation, including eIF4E [35] and Slf1p possesses a putative eIF4E-binding motif (YXXXXLϕ). Thus, it is tempting to speculate that Slf1p promotes translation of a subset of Puf3p targets and/or perhaps other nuclear transcripts that encode mitochondrial proteins to modulate mitochondrial respiration under certain conditions.

We have shown that extension of yeast chronological life span (CLS) by reduced TORC1 signaling requires mitochondrial respiration and involves increased mitochondrial translation and OXPHOS complex density [26], [27]. Even though respiration and OXPHOS complex density vary in response to altered Puf3p levels (Figures 1 and 3), we observed no changes in CLS in either wild-type or *tor1Δ* strains deleted for *PUF3* or in a wild-type strain over-expressing Puf3p (Figure 4A and 4B). However, over-expression of Puf3p reduces CLS in a *tor1Δ* strain (Figure 4C), but does not eliminate CLS extension completely. The two conclusions we draw from these results are 1) that changes in respiration induced by deletion of Puf3p must be of a different quality than that induced by reduced TORC1 signaling that leads to CLS extension and 2) that increased mitochondrial translation and respiration in a *tor1Δ* strain is necessary, but not sufficient for full CLS extension (i.e. over-expression of Puf3p provided a direct test of the requirement of these parameters in CLS extension by reduced TORC1 signaling). In this regard it is noteworthy that we recently showed that extension of CLS by TORC1 involves the generation of an adaptive mitochondrial ROS signal during growth (Pan et al. *Cell Metabolism*, in press). That this signal is not generated even though respiration is up-regulated in *puf3Δ* strains may explain why they do not have extended CLS (i.e. potentially explaining why increased respiration is necessary, but not sufficient to observe affects on CLS). Finally, over-expression of Slf1p increased median CLS (Figure 7D), which was accompanied by a moderate increase in respiration. Whether these effects of Slf1p are mediated by salient changes in respiration or cytoplasmic translation, which also contribute to aging [36], remains to be determined. It is tempting to speculate that these two processes may conspire to affect CLS, with alterations in cytoplasmic translation regulating the relative rate of translation of nuclear transcripts encoding mitochondrial proteins and hence altering mitochondrial gene expression and respiratory function in ways that influence the quality and quantity of respiration. Dissecting which aspects of increased respiration are beneficial to CLS and how they are regulated remains an important area of future investigation.

## Materials and Methods

### Yeast Strains

All experiments were performed using the strain background DBY2006 (*MATa his3-Δ200 leu2-3,-112 ura3-52 trp1-Δ1 ade2-1*) in synthetic dextrose (SD) media with appropriate amino acid supplements. Overnight 5 mL cultures were used to inoculate a 50 mL culture to a final OD_600_ of 0.1. Where indicated, strains were treated with 80 nM rapamycin (Sigma) or an appropriate volume of vehicle (ethanol) at an OD of 1.0 and assayed after the culture reached an OD of 2.5-3.0. Deletion of the *PUF3* ORF was achieved by transformation of DBY2006 with a *puf3::kanMx4* amplicon derived using PCR primers flanking the *PUF3* ORF and genomic DNA from the Open Biosystems *PUF3* knockout strain. To generate single and double knock-outs of *TOR1* and *PUF3*, wild-type and *tor1::kanMx4* strains were transformed with a *puf3::ura3* PCR amplicon derived using pRS316 as template and primers F (5′-GTT TCT TCT TTA AGC GCC CTG TCC CAT AGT AAC AAC AAC GAG ATT GTA CTG AGA GTG CAC-3′) and R (5′-GTT TCT CAA CAC TGG CTA AAT GTC TAT TTC CTA GGG AGT TCT GTG CGG TAT TTC ACA CCG-3′). In the strains used in Figure 2, the *PUF3* locus was replaced with *TRP1* using the primers stated above and pRS314 as a template, while the wild-type strain was made congenic by replacing the *ade2* locus with pRS314-derived *TRP1* using the primers F (5′-GCA AAC AGG CTC AAC ATT AAG ACG GTA ATA CTA GAT GCT GAG ATT GTA CTG AGA GTG CAC-3′) and R (5′-TCT TCT TCT TGC TTT AAT AAA AAC TGT TCC ATT TTC GTT GCT GTG CGG TAT TTC ACA CCG-3′). The *PUF3* ORF was amplified with the primers F (5′-GCT CAG CTG CAG TAC GGT AAT TGC GCA CTC TG-3′) and R (5′-GCT CAG GGA TCC CAT GAC AAC AGT ATT CTC AGT CAA A-3′) and cloned into the BamHI/PstI site of the plasmid YEp351. The *GSM1* ORF was amplified with the primers F (5′-AAT TAT AAG CTT ATC TCT GAC TGC CGT TTT GC-3′) and R (5′-AAT TAT AAG CTT TTG CCA ATC GGA CAA CAC TA-3′) and cloned into the HindIII site of YEp351. The *SLF1* ORF was amplified with the primers F (5′-ATT TAT CCC GGG TGC AGT ATG TTA TTG ATC CAT CG-3′) and R (5′-ATA AAT CCC GGG CTC TGG TGG CAT AAA ACA T-3′) and cloned into the XmaI site of YEp351.

### Chronological Life Span and Respiration Assays

Chronological life span was assayed as described previously [26]. 100 µl of culture was incubated with 100 µl of 4 mg/ml trypan blue for 5 minutes at 30°, and blue and clear cells were counted with a hemocytometer. Mitochondrial oxygen consumption was tested as described previously [26]. Briefly, 5 mL of yeast cultures were assayed at indicated stages of growth with a Clark-type electrode (model 5300A; YSI Bioanalytical). Addition of sodium azide to cultures ensured oxygen consumption was due to mitochondrial function.

### Transcript analysis by quantitative real-time PCR

RNA extraction and precipitation was carried out as described (Bonawitz et al. 2008) and purified further by RNeasy column (Qiagen). Using this RNA, cDNA was synthesized as described [37], using 3.2 µl of 50 µM OligodT_23_ and 2 µl of 40 µM random hexamer in a 20 µl reaction volume. The diluted cDNA was then mixed with 0.5 µl of 25 µM of each primer and 12.5 µl SYBR green [38] in a total volume of 25 µl and PCR performed using a Biorad C1000 thermocycler/CFX96 RT system used the following program: 1 cycle of 3 min at 95°C, followed by 35 cycles of 30 s at 95°C, 30 s at 53°C, and 30 s at 72°C. C_t_ values were obtained with Biorad CFX manger software and transcript levels were normalized using the actin control. The primers used were:

Actin RT F 5′-CCCAGGTATTGCCGAAAGAATGC-3′

Actin RT R 5′-GGAAGATGGAGCCAAAGCGG-3′;

Cox17 RT F 5′-CCAGAAAAGGAGGAGCGGGATA-3′

Cox17 RT R 5′-CGAAGCCATAACCCTTCATGCAC-3′;

Cox12 RT F 5′-CCCCAACAAAACCAAACAAAGCA-3′

Cox12 RT R 5′-TCCAAAAGACCTTGCACGGAG-3′;

Rip1 RT F 5′-TGGTCGGTGCTATGGGTCTTT-3′

Rip1 RT R 5′-AACATCGGCAGTAGCGGTCA-3′;

Slf1 RT F 5′-GGAAATTGCCCTTGGAAGCA-3′

Slf1 RT R 5′- GCCCAATTTTCGCGCCTAAT-3′.

### Western Immunoblotting

Protein extracts were prepared by TCA precipitation, separated on SDS-PAGE gels and transferred to PVDF (Millipore) membranes as described [39]. Antibodies directed against Cox2p (Invitrogen, 459150), Cox4p (Molecular Probes, A-6432), porin (Invitrogen, 459500), and actin (Chemicon) were diluted 1∶1000, incubated overnight, washed, and probed with goat-anti-mouse secondary diluted 1∶5000. Antibodies versus Coq5p, Pet100p, and Slf1p were obtained from colleagues and utilized as described above but diluted 1∶1000-1∶5000 and probed with donkey-anti-rabbit (Santa Cruz) secondary diluted by 1∶5000.

### Mitochondrial Translation Assays

Radiolabeling of mtDNA-encoded proteins was performed as described [40]. Briefly, cells that reached an OD of ∼1.0 were treated with 50 µl of 25 mg/ml cyclohexamide for 5 minutes and pulsed with 50 µCi of EXPRE^35^S^35^S Protein Labeling Mix (PerkinElmer) for 15 minutes. Cells were then washed, lysed using glass beads, and the resulting crude mitochondrial extracts were acquired by differential centrifugation. Protein (74 µg) was resolved on Any-kD precast gradient gels (BioRad). Gels were then dried, subjected to autoradiography, followed by rehydration and staining with coomassie blue.

### Relative mtDNA copy number

The amount of mtDNA per cell was determined as described previously [38]. Briefly, DNA was isolated using the phenol-chloroform “smash-and-grab” method, followed by incubation in 100 µl TE with 10 µg/mL RNase for 1 hour at 65°. DNA was diluted with water 1∶200-1∶400 and added to a 25 µl reaction mixture consisting of 14 µl of SYBR green and 0.5 µl of 16 µM forward and reverse primer. PCR and subsequent analysis were performed using the conditions described above for real-time PCR. The primers used were:

Cox1 RT F 5′-CTACAGATACAGCATTTCCAAGA-3′

Cox1 RT R 5′-GTGCCTGAATAGATGATAATGGT-3′;

Actin RT F 5′-GTATGTGTAAAGCCGGTTTTG-3′

Actin RT R 5′-CATGATACCTTGGTGTCTTGG-3′.
